# Dual Role of Dietary Curcumin Through Attenuating AFB_1_-Induced Oxidative Stress and Liver Injury via Modulating Liver Phase-I and Phase-II Enzymes Involved in AFB_1_ Bioactivation and Detoxification

**DOI:** 10.3389/fphar.2018.00554

**Published:** 2018-05-25

**Authors:** Ishfaq Muhammad, He Wang, Xiaoqi Sun, Xinghe Wang, Meiyu Han, Ziyin Lu, Ping Cheng, Muhammad A. Hussain, Xiuying Zhang

**Affiliations:** ^1^Heilongjiang Key Laboratory for Animal Disease Control and Pharmaceutical Development, Faculty of Basic Veterinary Science, College of Veterinary Medicine, Northeast Agricultural University, Harbin, China; ^2^Laboratory of Veterinary Pathology, Faculty of Basic Veterinary Science, College of Animal Science and Veterinary Medicine, Shenyang Agricultural University, Shenyang, China; ^3^Changchun Dirui Medical Company Ltd., Changchun, China; ^4^College of Life Science Engineering, Shenyang Institute of Technology, Fushun, China; ^5^College of Food Science, Northeast Agricultural University, Harbin, China

**Keywords:** CYP enzymes, Nrf2, AFB_1_, curcumin, liver

## Abstract

It is well understood that liver cytochrome p450 enzymes are responsible for AFB_1_ bioactivation, while phase-II enzymes regulated by the transcription factor nuclear factor-erythroid-2-related factor 2 (Nrf2) are involved in detoxification of AFB_1_. In this study, we explored the potential of curcumin to prevent AFB_1_-induced liver injury by modulating liver phase-I and phase-II enzymes along with Nrf2 involved in AFB_1_ bioactivation and detoxification. Arbor Acres broiler were divided into four groups including control group (G1; fed only basal feed), curcumin alone-treated group (G2; 450 mg/kg feed), AFB_1_-fed group (G3; 5 mg/kg feed), and curcumin plus AFB_1_ group (G4; 5 mg AFB_1_+450 mg curcumin/kg feed). After 28 days, liver and blood samples were collected for different analyses. Histological and phenotypic results revealed that AFB_1_-induced liver injury was partially ameliorated by curcumin supplementation. Compared to AFB_1_ alone-treated group, serum biochemical parameters and liver antioxidant status showed that curcumin supplementation significantly prevented AFB_1_-induced liver injury. RT-PCR and western blot results revealed that curcumin inhibited CYP enzymes-mediated bioactivation of AFB_1_ at mRNA and protein level. Transcription factor Nrf2, its downstream genes such as GSTA3, and GSTM2 mRNA, and protein expression level significantly upregulated via dietary curcumin. In addition, GSTs enzyme activity was enhanced with dietary curcumin which plays a crucial role in AFB_1_-detoxification. Conclusively, the study provided a scientific basis for the use of curcumin in broiler’s diet and contributed to explore the multi-target preventive actions of curcumin against AFB_1_-induced liver injury through the modulation of phase-I and phase-II enzymes, and its potent anti-oxidative effects.

## Introduction

Recently, several reports demonstrated that aflatoxin B_1_ (AFB_1_) is probably the most common researched mycotoxin in the world (Liu and Wu, 2010; Abrar et al., 2013). Many *Aspergillus* species such as *Aspergillus flavus* and *Aspergillus parasiticus* produce AFB_1_ (Nakai et al., 2008). It is considered to be one of the most harmful and potent aflatoxins among all other known mycotoxins (Ferenčík and Ebringer, 2003). AFB_1_ causes many adverse effects such as mutagenicity, carcinogenicity, hepatotoxicity, and immunotoxicity in humans, poultry and in many other animal species (Stettler and Sengstag, 2001; Kalpana et al., 2012). AFB_1_ has the ability to affect more than one system at a time and cause serious alterations in the normal homeostasis of the affected organism (Ferenčík and Ebringer, 2003; Herzallah, 2013). It has been placed in Group-1 human carcinogen by the International Agency for Research on Cancer (IARC) (Zuckerman, 2006). AFB_1_ leads to carcinogenicity in humans by p53 gene mutation at codon 249 and formation of DNA adducts (Aguilar et al., 1993, 1994). Previous reports showed that the liver is the major target organ of AFB_1_, as the liver plays a crucial role in drug or toxin metabolism and detoxification in the body (Grant et al., 2001; Rawal et al., 2010). AFB_1_ causes liver toxicity including infiltration of mononuclear and heterophilic cells and proliferation of bile duct along with changes in liver morphology, such as increase in liver weight, enlargement, congestion, necrosis, and pallor discoloration (Newberne and Butler, 1969; Hussain and Anwar, 2008), but the susceptibility of a species to AFB_1_ depends on various factors such as type of breed, immunity and nutritional status, age, sex, dose, length of exposure to AFB_1_, and managemental conditions (Richard, 2007).

The use of naturally occurring phytochemicals to combat human diseases and prevent toxicity is of growing scientific and public interest due to their potential therapeutic uses (Sharma et al., 2005). Curcumin is a polyphenol commonly composed of three types of curcuminoids, bisdemethoxycurcumin, demethoxycurcumin, and diferuloylmethane, derived from the rhizome of *Curcuma longa* (Common name: turmeric) famous for its pharmacological and therapeutic properties (Anand et al., 2008). Several reports have documented the anti-inflammatory, anti-oxidative, anti-cancerous, and potent chemotherapeutic properties of curcumin (Aggarwal et al., 2003; Joe et al., 2004; Liew and Mohd-Redzwan, 2018; Limaye et al., 2018; Rauf et al., 2018). It has been proved earlier that curcumin is effective against AFB_1_-induced oxidative stress (Gowda et al., 2009; El-Bahr, 2015). Moreover, previous studies demonstrated that curcumin modulated phase-I enzymes activity and has the potential to control AFB_1_-induced carcinogenesis and toxic effects (Firozi et al., 1996; Appiah-Opong et al., 2007; Nayak and Sashidhar, 2010). Cytochrome p450 (phase-I) enzymes are responsible for the biotransformation of AFB_1_ into its harmful metabolites (Yunus et al., 2011). Earlier reports showed that several CYP enzymes (CYP1A1, CYP1A2, CYP1A5, CYP2A6, CYP3A37, and CYP3A4) were involved in bioactivation of AFB_1_ into its harmful electrophilic metabolite aflatoxin-8,9-epoxide (AFBO) and to AFM_1_, AFP_1_, AFQ_1_, etc. (Gregorio et al., 2015). AFBO, the most potent metabolite may quickly react with DNA to form DNA adducts or cause mutations (Aguilar et al., 1993), or conjugated with phase-II enzymes such as glutathione S-transferases (GST) (Johnson et al., 2014). Importantly, the sensitivity of a species to AFB_1_ is not only related to AFB_1_ bioactivation via CYP enzymes but also related to detoxification enzymes (phase-II enzymes) (Klein et al., 2000). It is well known that the transcription factor nuclear factor-erythroid-2-related factor 2 (Nrf2) plays a crucial role in regulating several pathways, including drug metabolism, antioxidant, and detoxification such as phase-II enzymes like superoxide dismutase, catalase, NAD(P)H:quinine oxidoreductase-1, uridine 5-diphospho (UDP) glucuronosyltransferase, heme oxygenase-1 (HO-1), glutathione *S*-transferase, glutathione peroxidase (GPx), thioredoxin, and uridine 5-diphospho (UDP)-glucuronosyltransferase (Li et al., 2008). Besides, Slocum and Kensler’s (2011) studies demonstrated that up-regulation of Nrf2-pathway and its downstream genes (GSTs) prevented chemical carcinogenesis . Moreover, Nrf2 plays an important cytoprotective role in disease prevention, especially at the initiation stage of cancer development (Krajka-Kuźniak et al., 2017). Thus, inhibition of phase-I enzymes (involved in AFB_1_-bioactivation) and activation of Nrf2-pathway and its downstream genes (related to AFB_1_-detoxification) could be a novel pharmacological approach for the prevention of AFB_1_-induced liver injury and oxidative stress in broilers. The present study was designed to shed light on the multi-target effects of curcumin through inhibition of phase-I enzymes, and activation of Nrf2 as well as phase-II enzymes against AFB_1_-induced liver injury and ameliorating oxidative stress in Arbor Acres broiler liver. The study contributed to provide a biochemical basis for the supplementation of curcumin in broiler’s diet along with its chemopreventive action against AFB_1_-induced liver injury.

## Materials and Methods

### Ethics Statement

All the experiments were performed according to animal ethics guidelines and approved protocols of the Harbin Veterinary Research Institute of the Chinese Academy of Agricultural Sciences [SYXK (Hei) 2012-2067].

### Chemicals and Reagents

Methanol, ethanol (99.0%), isopropanol, chloroform, and glycine were bought from Tian Li Company Ltd. (Tianjin, China). AFB_1_ (purity ≥ 99.0%) was obtained from Sigma-Aldrich (St. Louis, United States). Curcumin (2.5%) was bought from Weisheng Long Chemical Company Ltd. (China). Hematoxylin and eosin were bought from Nanjing chemical reagent factory (China).

### Experimental Chickens, Diets, and Sample Collection

One-day-old healthy Arbor Acres broiler chickens were bought from Yi Nong commercial hatchery (Registration number; 230108799294096, Heilongjiang, China). The broilers were divided in four groups (15 chicks/group) and assigned to different cages. Chickens were provided with 12 h light and 12 h dark cycle and fed *ad libitum* for 28 days. Four dietary groups were: (G1) control group; fed only basal diet, (G2) curcumin control group: basal diet incorporated with 450 mg curcumin/kg feed; (G3) AFB_1_ group: basal diet contaminated with 5.0 mg AFB_1_/kg feed; and (G4) curcumin+AFB_1_ group: basal diet incorporated with 5.0 mg AFB_1_ and 450 mg curcumin/kg feed. After 28 days, chickens were first weighed, euthanized with sodium pentobarbital, and sacrificed in an antiseptic environment. Liver was collected, weighed and stored at -80°C for further analysis.

### Serum Biochemical Analysis

Serum biochemical markers were measured to evaluate liver toxicity. 5 ml blood sample was collected from the vein wing before slaughtering of chickens. Serum was separated after centrifugation at 3000 *g* for 1.5 h and biochemical analysis was carried out at the same time. Serum samples were analyzed for alanine aminotransferase (ALT), aspartate aminotransferase (AST), alkaline phosphatase (ALP), and gamma-glutamyl transferase (GGT) activities in an automatic biochemical analyzer (Model 7080, Japan Hitachi Limited).

### Histological Observation

Liver morphology was assessed by histopathological examination. Fresh liver tissue pieces were stained with hematoxylin and eosin (HE) as previously described (Wang X.H. et al., 2016). In brief, liver sections (1 cm × 1 cm) were fixed in 10% formalin for 24 h. The fixed liver tissues were embedded in paraffin after processed through a series of graded ethanol and dimethyl benzene. 4-μm tissue sections were mounted on glass slides, and finally stained with HE dye for microscopic examination (Light microscope; Nikon E100, 40× magnification).

### Measurement of Antioxidant Enzyme Activities

A piece of fresh liver tissue (0.4–0.5 g) was immediately taken out after slaughtering and chilled in 0.85% NaCl at 0°C. It was then dried and homogenized as previously described (Soetikno et al., 2013). The homogenized solution was then centrifuged (12,000 ×*g* for 15 min) at 4°C and the supernatants were analyzed for antioxidant activity. All the kits were bought from Nanjing Jiancheng Bioengineering Factory (China). GSH-Px (Glutathione peroxidase, kit; A005), SOD (Superoxide dismutase, kit; A001-1) and MDA (Malonaldehyde, kit; A003-1) activities were measured in the supernatant according to the instructions of the kit.

### RNA Extraction and Real-Time Quantitative Polymerase Chain Reaction (qPCR)

TRIzol (Invitrogen Inc., Carlsbad, CA) reagent was used to isolate total RNA (Cheng et al., 2017). RNA quality was verified by measuring the 260/280 ratio (Wang J. et al., 2016). Toyobo first strand cDNA synthesis kit (Osaka, Japan) was used for the synthesis of cDNA. The target genes and β-actin loading control gene specific primers are given in **Table 1**. The kit bought from Toyobo Company Ltd. (Japan) was utilized for RT-PCR reaction in a Roche LightCycle instrument (Shanghai, China). The data were analyzed according to 2^-ΔΔC_t_^ method (Livak and Schmittgen, 2001).

**Table 1:** Primers used for quantitative real-time PCR.

Accession no.	Target gene	Primer	Sequence (5′–3′)	Length (bp)
NM_205147.1	CYP1A1	Forward	GAAGGGACCGAAGTGAACAA	115
		Reverse	TGGACAGGAAAAGGAACACC	
NM_205146.2	CYP1A2	Forward	CATGTACCTTGTGACGCAGC	158
		Reverse	CAAATTGCCAGGTCGGAACC	
KX687985.1	CYP2A6	Forward	CTGCAGAGAATGGCATGAAG	110
		Reverse	CCTGCAAGACTGCAAGGAA	
NM_001329508.1	CYP3A4	Forward	AGTGGAGTTCAATGGGGTGA	160
		Reverse	CAGGAAGGTGTAGGGGTCAA	
NM_205117.1	Nrf2	Forward	GATGTCACCCTGCCCTTAGA	124
		Reverse	TCGTTCCATTTGTTCCTTCTG	
NM_001001777.1		Forward	AGACCAGAGCCATCCTCAAC	
	GSTA3,	Reverse	TGCCAGTCCTTCCACATACA	101
NM_205090.1		Forward	CCCAACCTGCCCTATCTCAT	
	GSTM2	Reverse	CTGCTTCTCCACCTCCGTCT	114
X00182.1		Forward	TGAAGCCCAGAGCAAAAGAG	
	β-actin	Reverse	TGCTCCTCAGGGCTACTCTC	135


### Western Blot Analysis

Western blotting was performed as described previously (Lu et al., 2017). In brief, fresh liver tissues were homogenized in RIPA and PMSF. 5 × SDS-PAGE loading buffer was added, followed by protein degeneration via boiling for 5 min. The loaded protein were separated by 10 or 12% SDS-PAGE, and transferred to nitrocellulose membrane (NC). The membranes were incubated for 1 h in non-fat dry milk in TBST. CYP1A1 (1:500 diluted, Sangon Biotech. Shanghai, China), CYP1A2 (1:400 diluted, Sangon Biotech, Shanghai, China), CYP3A4 (1:200 diluted, Sangon biotech. Shanghai, China), CYP2A6 (1:200 diluted, ImmunoWay Biotechnology Company, Suzhou, China), Nrf2 (1:500 diluted, Wanleibio Co., Ltd., Harbin, China), GSTA3, (1:200 diluted, Life Span Biosciences, Inc., Shanghai, China), GSTM2 (1:800 diluted, BBI Life Sciences, Canada), and β-actin (1:1000 diluted, Applied Biological Materials Inc., Canada) or GAPDH (1:100 diluted, Applied Biological Materials Inc., Canada) protein was detected using specific primary antibodies incubated overnight at 4°C. Secondary anti-mouse or anti-rabbit horseradish peroxidase conjugated IgG was used for 1 h, and immune-complexes were detected using enhanced chemiluminescence (ECL) detection. Signals were visualized on a computer using Image J software (National Institute of Health, United States).

### Statistical Analysis

The data significance were analyzed by one way ANOVA followed by LSD test using the statistical package for social science (SPSS, version 21.0), and *p* < 0.05 was considered statistically significant. The experiments were performed at least three times unless otherwise mentioned. The data are represented as means ± standard errors. GraphPad Prism (Version 6.01) was used to draw all the graphs with standard error bars.

## Results

### Curcumin Prevented AFB_1_-Induced Liver Injury

**FIGURE 1:**
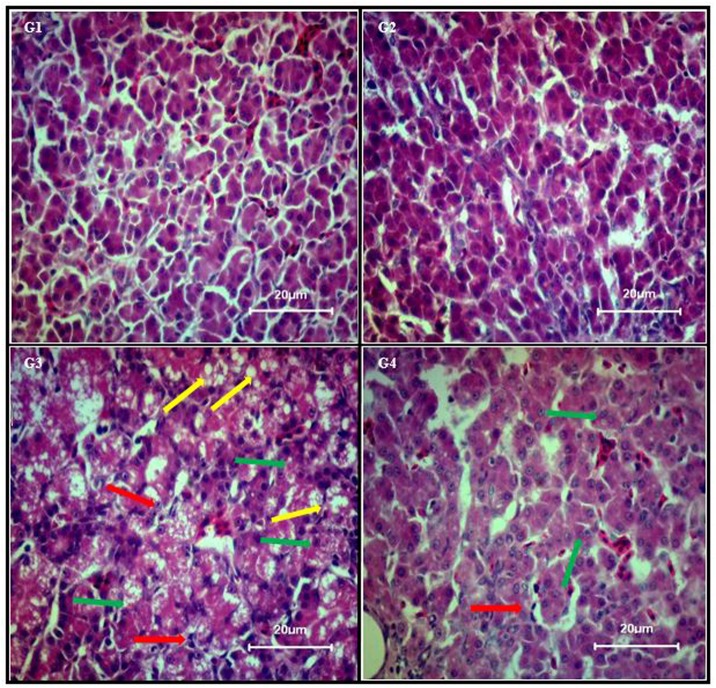
Chicken liver photomicrographs of hematoxylin and eosin-stained liver sections observed under light microscope (40X magnification), where n = 3 (H&E, scale bar = 20 μm). Treatments represented as **(G1)** control group; fed only basal diet, **(G2)** curcumin control group; basal diet + 450 mg curcumin/kg feed, **(G3)** AFB_1_ group; basal diet + 5.0 mg AFB_1_/kg feed, and **(G4)** curcumin+AFB_1_ group; basal diet + 5.0 mg AFB_1_ + 450 mg curcumin/kg feed. Yellow arrow, hepatic steatosis; red arrow, lymphocyte infiltration; and green arrow, hepatic granular degeneration.

Liver sections from control group (**Figure 1**; G1) and curcumin control group (**Figure 1**; G2) showed normal morphology. Dietary AFB_1_ induced severe liver injury (**Figure 1**; G3) compared to control and curcumin control group. Severe to moderate lesions such as hepatic granular degeneration, lymphocyte infiltration, and hepatic steatosis were observed in AFB_1_-fed group. The supplementation of curcumin in the diet partially ameliorated AFB_1_-induced hepatic lesions (**Figure 1**; G4). These results confirmed the preventive effects of curcumin against AFB_1_-induced liver injury.

### Effect of Curcumin and AFB_1_ on Serum Biochemical Parameters and Body and Liver Weight

Serum biochemical parameters (ALP, ALT, AST, and GGT level) are represented in **Table 2**. Compared to control group (**Table 2**; G1), AFB_1_ supplemented diet (**Table 2**; G3) significantly (*p* < 0.05) affected serum ALP, ALT, AST, and GGT level, While compared to curcumin control group (**Table 2**; G2), AFB_1_-induce increase in ALT, ALP, and AST level is not statistically significant (*p* > 0.05) except GGT level (*p* < 0.05) (**Table 2**; G3). However, curcumin supplementation in the diet prevented AFB_1_-induced changes in serum biochemical parameters (**Table 2**; G4). The changes in serum biochemical parameters were inconsistent with the liver histological analysis (**Figure 1**). In addition, body weight and liver weight were recorded to study the changes in phenotypic effects (**Table 2**). Notably, compared to control (**Table 2**; G1) and curcumin control group (**Table 2**; G2), body weight significantly (*p* < 0.05) decreased in AFB_1_-fed group (**Table 2**; G3). Moreover, liver weight significantly (*p* < 0.05) decreased in AFB_1_-fed group. However, the supplementation of dietary curcumin (**Table 2**; G4) reversed AFB_1_-induced changes in body and liver weight significantly (*p* < 0.05).

**Table 2:** Effect of curcumin and AFB_1_ on serum biochemical parameters, liver weight, and body weight of chickens.

Name Groups	ALP (IU/L)	ALT (IU/L)	AST (IU/L)	GGT (IU/L)	Liver weight (g/bird)	Body weight (g/bird)
G1	1725.00 ± 13.75	1.25 ± 0.03	209.53 ± 1.54	21.00 ± 0.14	21.37 ± 1.38	991.58 ± 19.73
G2	1703.67 ± 14.27	1.50 ± 0.04	219.50 ± 1.37	22.00 ± 0.13	17.32 ± 0.86	873.36 ± 12.92^∗^
G3	2446.33 ± 14.3^∗^	3.75 ± 0.06^∗^	262.0 ± 1.16^∗^	35.00 ± 0.25^∗,∗∗^	28.49 ± 1.74^∗∗^	608.00 ± 21.43^∗,∗∗^
G4	1540.50 ± 28.73^∗∗∗^	2.00 ± 0.02^∗∗∗^	196.5 ± 1.78^∗∗∗^	15.33 ± 0.19^∗∗∗^	20.03 ± 0.63^∗∗∗^	823.00 ± 16.02^∗,∗∗∗^


**Table 1** shows serum ALP, ALT, AST, GGT activities (*n* = 3), and liver weight (grams) and body weight (grams) (*n* = 5). The values represented as mean ± SE. ^∗^*p* < 0.05 vs. control group (G1), ^∗∗^*p* < 0.05 vs. curcumin control group (G2) and ^∗∗∗^*p* < 0.05 vs. AFB1 group (G3) according to LSD test. Treatments represented as (G1) control group; fed only basal diet, (G2) curcumin control group; basal diet + 450 mg curcumin/kg feed, (G3) AFB_*1*_ group; basal diet + 5.0 mg AFB_*1*_/kg feed and (G4) curcumin+AFB_*1*_ group; basal diet + 5.0 mg AFB_*1*_ + 450 mg curcumin/kg feed.

### Curcumin Attenuated AFB_1_-Induced Oxidative Stress

**FIGURE 2:**
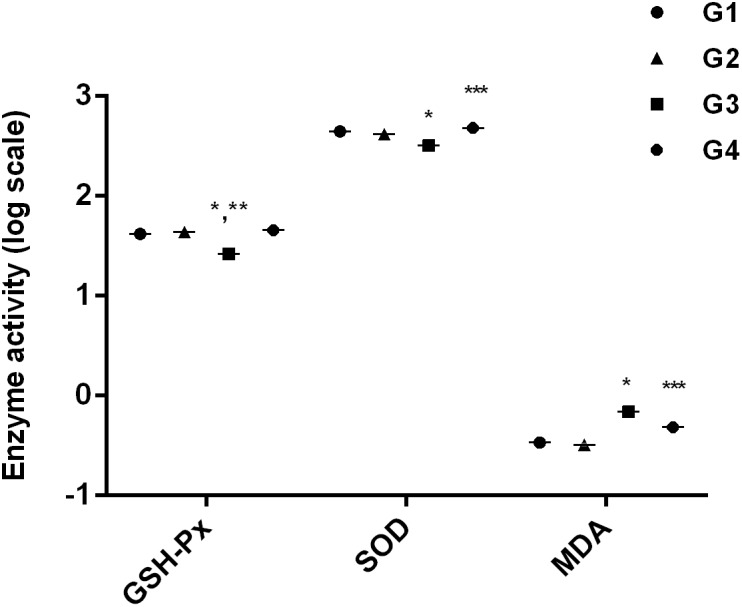
It represents anti-oxidant activities of GSH-Px activity, SOD activity, and MDA activity. The values are represented as mean ± SE (*n* = 5). ^∗^*p* < 0.05 vs. control group (G1), ^∗∗^*p* < 0.05 vs. curcumin control group (G2), and ^∗∗∗^*p* < 0.05 vs. AFB1 group (G3). Treatments represented as (G1) control group: fed only basal diet, (G2) curcumin control group: basal diet + 450 mg curcumin/kg feed, (G3) AFB_1_ group: basal diet + 5.0 mg AFB_1_/kg feed, and (G4) curcumin+AFB_1_ group: basal diet + 5.0 mg AFB_1_ + 450 mg curcumin/kg feed.

After 28 days, the hepatic antioxidant parameters were significantly (*p* < 0.05) altered by dietary AFB_1_ (**Figure 2**). Compared to control group (**Figure 2**; G1), GSH-Px and SOD activity were significantly (*p* < 0.05) decreased while MDA activity was significantly (*p* < 0.05) enhanced in AFB_1_-fed group (**Figure 2**; G3). The addition of curcumin alone (**Figure 2**; G2) increased GSH-Px activity significantly (*p* < 0.05) but not significantly affected SOD and MDA activity. Importantly, dietary curcumin (**Figure 2**; G4) significantly (*p* < 0.05) reversed AFB_1_-induced changes in antioxidant parameters. It is obvious from the results that curcumin effectively ameliorated AFB_1_-induced stress in broiler liver.

### Inhibition of Phase-I Enzymes

**FIGURE 3:**
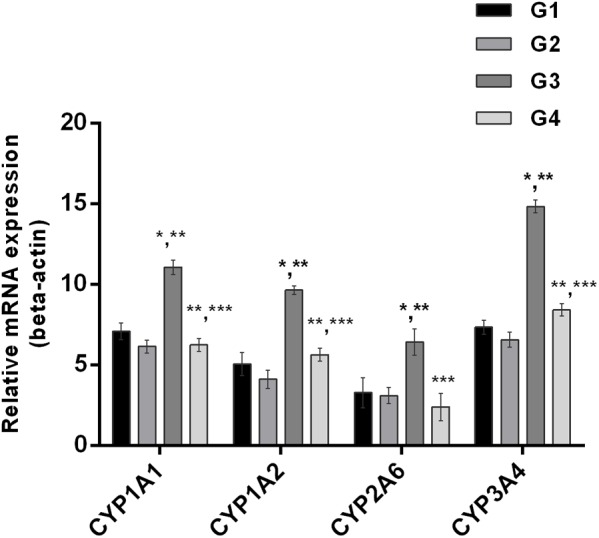
Figure shows the liver mRNA expression of CYP1A1, CYP1A2, CYP3A4, and CYP2A6 enzyme of the four treated groups. The values are represented as mean ± SE (*n* = 3). Treatments represented as (G1) control group: fed only basal diet, (G2) curcumin control group: basal diet + 450 mg curcumin/kg feed, (G3) AFB_1_ group: basal diet + 5.0 mg AFB_1_/kg feed, and (G4) curcumin+AFB_1_ group: basal diet + 5.0 mg AFB_1_ + 450 mg curcumin/kg feed. The value of *P* < 0.05 was considered statistically significant. ^∗^*p* < 0.05 vs. control group (G1), ^∗∗^*p* < 0.05 vs. curcumin control group (G2) and ^∗∗∗^*p* < 0.05 vs. AFB1 group (G3).

**FIGURE 4:**
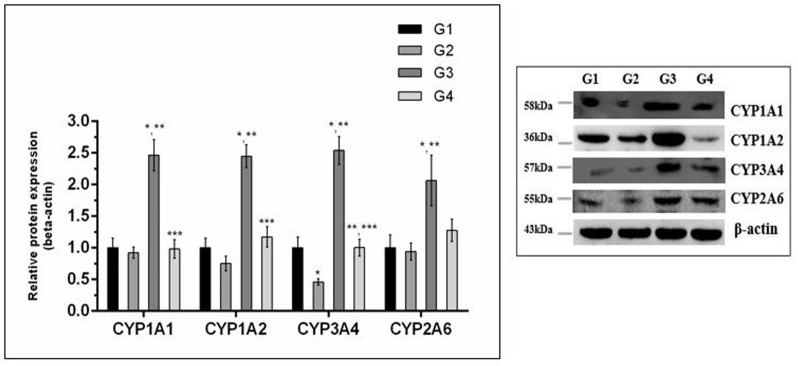
Western blot analysis of CYP enzymes involved in AFB_1_-bioactivation. CYP1A1, CYP1A2, CYP3A4, and CYP2A6 protein expression level of the four treated groups shown in the figure. The intensity of the protein bands was quantified by densitometry analysis. The levels of beta-actin were used as internal standard control. The values represented as mean ± SE (*n* = 3). Treatments represented as (G1) control group: fed only basal diet, (G2) curcumin control group: basal diet + 450 mg curcumin/kg feed, (G3) AFB_1_ group: basal diet + 5.0 mg AFB_1_/kg feed and (G4) curcumin+AFB_1_ group: basal diet + 5.0 mg AFB_1_ + 450 mg curcumin/kg feed. The value of *P* < 0.05 was considered statistically significant. ^∗^*p* < 0.05 vs. control group (G1), ^∗∗^*p* < 0.05 vs. curcumin control group (G2) and ^∗∗∗^*p* < 0.05 vs. AFB1 group (G3).

The effect of curcumin, AFB_1,_ and curcumin+AFB_1_ diet was investigated at the mRNA and protein level. **Figure 3** shows four major CYP enzymes (CYP1A1, CYP1A2, CYP3A4, and CYP2A6) mRNA expression levels. Compared to control (**Figure 3**; G1) and curcumin control group (**Figure 3**; G2), AFB_1_ (**Figure 3**; G3) significantly (*p* < 0.05) upregulated the mRNA expression level of the four CYP enzymes. However, curcumin supplementation (**Figure 3**; G4) in diet significantly (*p* < 0.05) down-regulated AFB_1_-induced increase in CYP enzymes. Protein expression levels (**Figure 4**) showed the same trends as mRNA expression levels. Compared to control (**Figure 4**; G1) and curcumin control group (**Figure 4**; G2), a significant (*p* < 0.05) increase has been noted in all CYP enzyme protein levels in AFB_1_-fed group (**Figure 4**; G3). While curcumin supplementation (**Figure 4**; G4) significantly (*p* < 0.05) decreased the protein expression levels of CYP1A1, CYP1A2 and CYP3A4, the decrease is not statistically significant for CYP2A6 (*p* > 0.05) protein expression. The data clearly showed that CYP1A1, CYP1A2, CYP3A4 and CYP2A6 actively involved in AFB_1_ bioactivation. However, curcumin supplementation significantly down-regulated the expression level of these enzymes at mRNA and protein level, and hence inhibited CYP enzymes-mediated bioactivation of AFB_1_.

### Curcumin Induced the Transcriptional Activation of Nrf2 and Phase-II Enzymes

**FIGURE 5:**
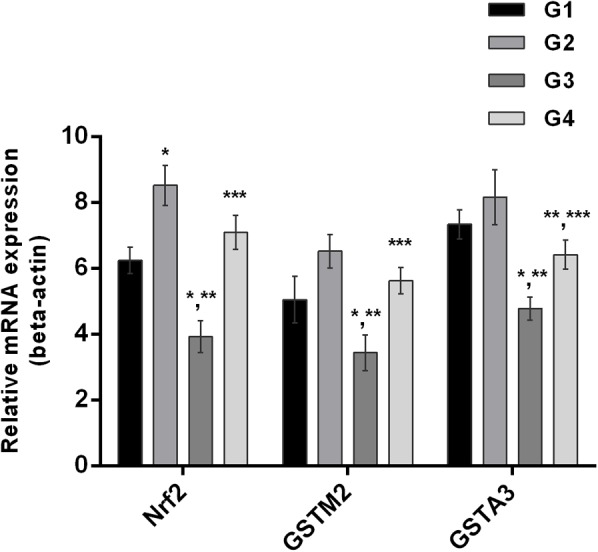
Nrf2, GSTA3, and GSTM2 mRNA expression level of the four treated groups shown in the figure. Treatments represented as (G1) control group: fed only basal diet, (G2) curcumin control group: basal diet + 450 mg curcumin/kg feed, (G3) AFB_1_ group: basal diet + 5.0 mg AFB_1_/kg feed and (G4) curcumin+AFB_1_ group: basal diet + 5.0 mg AFB_1_ + 450 mg curcumin/kg feed. The values represented as mean ± SE (*n* = 3). The value of *P* < 0.05 was considered statistically significant. ^∗^*p* < 0.05 vs. control group (G1), ^∗∗^*p* < 0.05 vs. curcumin control group (G2) and ^∗∗∗^*p* < 0.05 vs. AFB1 group (G3).

**FIGURE 6:**
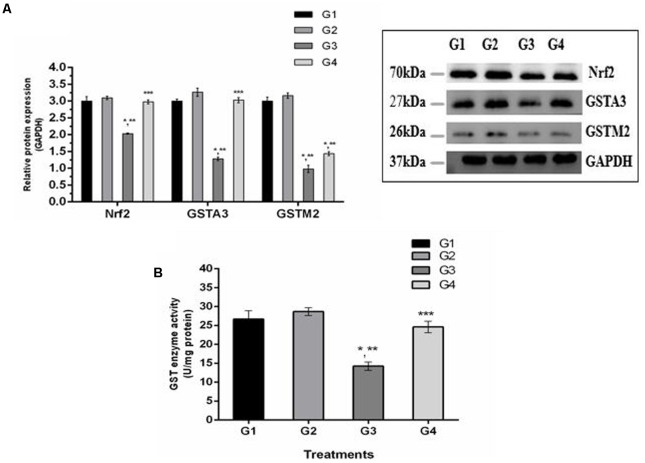
Western blot analysis of Nrf2, GSTA3, and GSTM2 involved in AFB_1_-detoxification. **(A)** Nrf2, GSTA3, and GSTM2 protein expression level of the four treated groups shown. The intensity of the protein bands was quantified by densitometry analysis. The levels of GAPDH gene were used as internal standard control. The values represented as mean ± SE (*n* = 3). **(B)** Cytosolic GST enzyme activity (U/mg protein) of the four treated groups presented in the figure. Treatments represented as (G1) control group: fed only basal diet, (G2) curcumin control group: basal diet + 450 mg curcumin/kg feed, (G3) AFB_1_ group: basal diet + 5.0 mg AFB_1_/kg feed and (G4) curcumin+AFB_1_ group: basal diet + 5.0 mg AFB_1_ + 450 mg curcumin/kg feed. The value of *P* < 0.05 was considered statistically significant. ^∗^*p* < 0.05 vs. control group (G1), ^∗∗^*p* < 0.05 vs. curcumin control group (G2) and ^∗∗∗^*p* < 0.05 vs. AFB1 group (G3).

It is well understood that Nrf2 plays a crucial role in the defense system of a body and is an important regulator of several cellular pathways including phase-II enzymes involved in the detoxification of AFB_1_. Here, we examined the changes induced by diets supplemented with AFB_1_ and curcumin alone or in combination on Nrf2, GSTA3, and GSTM2 at mRNA and protein level. The mRNA expression level of Nrf2, GSTA3, and GSTM2 are displayed in **Figure 5**. Compared to control (**Figure 5**; G1) and curcumin control group (**Figure 5**; G2), AFB_1_ (**Figure 5**; G3) significantly (*p* < 0.05) down-regulated Nrf2, GSTA3, and GSTM2 mRNA expression level. Interestingly, curcumin significantly (*p* < 0.05) enhanced AFB_1_-induced decrease in Nrf2, GSTA3, and GSTM2 mRNA expression level (**Figure 5**; G4). Similar trends were observed in Nrf2, GSTA3, and GSTM2 protein expression level (**Figure 6A**). Notably, Nrf2, GSTA3, and GSTM2 protein expression level has been significantly (*p* < 0.05) reduced in AFB_1_-fed group (**Figure 6A**; G3) relative to control (**Figure 6A**; G1) and curcumin control group (**Figure 6A**; G2). On the other hand, curcumin supplemented diet (**Figure 6A**; G4) significantly (*p* < 0.05) restored AFB_1_-induced decrease in Nrf2 and GSTA3, protein level. However, the increase in GSTM2 (**Figure 6A**; G4) is not statistically significant relative to AFB_1_-fed group (**Figure 6A**; G3). The data showed that curcumin significantly upregulated the transcription factor Nrf2 and subsequently its downstream genes such as phase-II enzymes (GSTA3, and GSTM2), and thus improves the defense system against AFB_1_-induced toxicity.

### Hepatic GST Enzyme Activity

GST enzymes play an important role in the detoxification (conjugation) of AFB_1_ metabolites. **Figure 6B** displays GST enzyme activity detected in liver cytosol. It has been noted that AFB_1_-supplemented diet (G3) significantly (*p* < 0.05) reduced GST enzyme activity compared to control group (**Figure 6B**; G1) and curcumin control group (**Figure 6B**; G2). However, GST activity was markedly (*p* < 0.05) elevated in curcumin+AFB_1_-fed group (**Figure 6B**; G4) relative to AFB_1_-fed group (**Figure 6B**; G3). These findings showed that curcumin actively increased GST enzyme activity to catalyze the harmful metabolites of AFB_1_.

## Discussion

Numerous studies reported that AFB_1_ is a well-known hepatotoxic and hepatocarcinogen and affects many systems at a time (Rawal et al., 2010). The current study showed that AFB_1_ significantly modulated serum biochemical parameters. Liver histological observations showed abnormal morphological signs in AFB_1_-treated group, while oxidative stress parameters clearly revealed that AFB_1_ induced oxidative stress in hepatocytes. Liver weight markedly increased and body weight was significantly suppressed in the current study in AFB_1_-fed group. These abnormal findings related to AFB_1_ toxicity were in general agreement with previous studies (Kumar and Balachandran, 2009; Ibrahim, 2013; El-Bahr, 2015; Muhammad et al., 2017). Notably, our data showed that dietary supplementation of curcumin ameliorated AFB_1_-induced oxidative stress and reversed serum biochemical changes. AFB_1_-induced abnormal morphological signs in the liver partially disappear with curcumin treatment. The above findings were in agreement with some earlier reports that curcumin significantly mitigated AFB_1_-induced toxicity in broiler liver (Gowda et al., 2008, 2009). It has been reported earlier that CYP1A1, CYP1A2, CYP2A6, and CYP3A4 are responsible for the bioactivation of AFB_1_ in poultry species (ducks, quails, turkeys and chickens) (Diaz et al., 2010a,b). In this study, liver CYP1A1, CYP1A2, CYP3A4, and CYP2A6 were significantly upregulated in AFB_1_-fed group showing its involvement in AFB_1_ biotransformation into harmful metabolites. Interestingly, the inhibition of these enzymes may be a promising treatment to prevent AFB_1_-induced liver toxicity. Importantly, the use of curcumin markedly reduced the mRNA and protein expression levels of the selected CYP enzymes in broiler liver *in vivo*. Our previous study demonstrated that curcumin inhibited CYP2A6 enzyme activity (in a dose dependent manner) involved in AFB_1_-bioactivation (Muhammad et al., 2017). Zhang et al. (2016) also reported that curcumin significantly inhibited CYP enzymes-mediated bioactivation of AFB_1_ into AFBO. Similarly, it has been noted that AFB_1_-induced increase expression of chicken CYP2H1 and CYP1A1 was alleviated with dietary curcumin (Yarru et al., 2009). Moreover, curcumin attenuated Benzo[a]Pyrene-induced oxidative stress and reduced DNA adducts in mice via inhibiting CYP1A1/1A2 activities (*in vivo*) (Garg et al., 2008), decreased CYP3A activity in rats (Kim et al., 2015), and modulated CYP1A2, CYP3A4, CYP2B6, CYP2C9, and CYP2D6 activity in humans (Appiah-Opong et al., 2007). Thus, curcumin could possibly prevent AFB_1_-induced hepatotoxicity though the synergistic effects of increased antioxidant capacities and inhibition of the cytochrome enzymes mediated activation to AFBO. However, the mechanism behind cytochrome P450 enzymes inhibition is still unknown and further molecular studies are needed to know the exact molecular mechanism of curcumin-mediated CYP450 inhibition.

Transcription factor Nrf2 is an attractive target in toxin chemoprevention due to its cytoprotective response and combat oxidative stress leading to prevention of DNA damage, carcinogenesis, and removal of toxic metabolites from the body (Slocum and Kensler, 2011). Previous reports demonstrated that curcumin is a potent inducer of Nrf2-pathway and its downstream genes involved in many cytoprotective mechanisms (Sahin et al., 2012; Soetikno et al., 2013). Nrf2 plays a crucial role in chemoprevention and reduce liver injury via up-regulating GST enzymes responsible for the detoxification of AFB_1_ harmful metabolites such as AFBO (Poapolathep et al., 2015), and meanwhile upregulate antioxidant defense system (Soetikno et al., 2013). The nuclear translocation of AFB_1_ is associated with many cellular proteins carrying signals for its translocation and subsequent binding to DNA, which results in DNA damage or causes deterioration of cellular proteins (Ricordy et al., 2002). Intriguingly, AFB_1_-induced impairment of specific protein functions or DNA damage decreased cell survival in cultured cells (Besaratinia et al., 2009). Previous studies demonstrated that protein biosynthesis is weakened due to the formation of AFB_1_-DNA adducts. In addition, AFB_1_ could change the activity of enzymes such as cyclic nucleotide phosphodiesterase, protein kinase, and Ca^2+^-ATPase, alter hepatic protein phosphorylation (Mistry et al., 1996; Ricordy et al., 2002), and causes p53 mutation which may develop carcinogenesis in experimental animals (Aguilar et al., 1994). Similarly, our data showed that Nrf2 mRNA and protein expression level significantly down-regulated in AFB_1_-fed group. Consequently, the downstream genes of Nrf2 such as GSTA3, and GSTM2 mRNA and protein expression levels markedly decreased in AFB_1_-fed group. Hence, it is speculated that the reduction in protein level could be due to AFB_1_-induced impairment in protein synthesis in broiler hepatocytes. Meanwhile, curcumin supplementation reversed AFB_1_-induced decrease in Nrf2, GSTA3, and GSTM2 mRNA and protein expression level. Furthermore, curcumin markedly increased GST enzyme activity involved in detoxification (conjugation) of AFB_1_. Similarly, previous studies reported that GST enzymes play a major role in prevention of AFB_1_-induced toxic effects and carcinogenesis (Larsson et al., 1994), and could prevent AFB_1_-induced hepatic carcinoma in fisher F344 rats via Nrf2-pathway (Eaton and Schaupp, 2014). We found that curcumin could inhibit cytochrome P450 enzymes-mediated bioactivation of AFB_1_ along with activation of Nrf2-pathway. In addition, our data showed that curcumin alleviated oxidative stress and caused alteration in AFB_1_-induced changes in serum biochemical parameters. These evidences suggested that curcumin could effectively inhibit AFB_1_-induced toxicity in broilers liver. Recently, many studies reported the chemopreventive effects of curcumin against AFB_1_-induced liver injury in several experimental animals (Larsson et al., 1994; Gowda et al., 2008; Yarru et al., 2009; Poapolathep et al., 2015; Zhang et al., 2016). However, these studies only investigated the preventive effects of curcumin through phase-I enzymes or phase-II enzymes against AFB_1_-induced liver injury. In contrast, in this study, we scrutinized the multi-target effects of curcumin regulating both phase-I enzymes and phase-II enzymes via Nrf2-pathway involved in AFB_1_ bioactivation and detoxification. It has been noted from our previous study that 450 mg/kg curcumin provided maximum amelioration and significantly inhibited CYP2A6-mediated AFB_1_ bioactivation (Muhammad et al., 2017). Therefore, in this study, we selected the dose of curcumin (450 mg/kg feed) to explore its preventive effects against dietary AFB_1_ at a dose of 5 mg/kg feed. However, previous studies reported that curcumin is regarded a safe compound at a dose of 12 g/day (Basak et al., 2015), acts as anti-inflammatory, anti-tumor, and anti-oxidant, etc. (Abdel-Wahhab, 2015), regulates pharmacological activities in a direct or indirect way and modulates various signaling molecules including cell survival proteins, apoptosis, drug resistance protein, etc. (Basak et al., 2015; Abdel-Wahhab et al., 2016), and possess the ability to scavenge free radicals (Trujillo et al., 2013). Our data also showed that curcumin prevented liver dysfunction due to its anti-oxidant activity as well as modulating drug metabolizing enzymes including liver phase-I enzymes, Nrf2, and phase-II enzymes. In addition, higher studies are needed to investigate the preventive effects of curcumin and AFB_1_-induced undesirable changes in other cellular pathways such as cell cycle arrest, autophagy, apoptosis and cell death.

## Conclusion

In conclusion, we showed that dietary AFB_1_ negatively altered serum biochemical parameters, reduced activities of liver anti-oxidative enzymes, suppressed Nrf2 and phase-II enzymes involved in AFB_1_ detoxification, and markedly increased the expression level of CYP enzymes involved in AFB_1_ biotransformation, while dietary curcumin significantly reversed AFB_1_-induced alterations in serum biochemical parameters and enhanced liver antioxidant enzyme activities. Moreover, curcumin inhibited CYP enzymes-mediated bioactivation of AFB_1_ and upregulated Nrf2 defense system including GST enzyme activity. Therefore, the above novel findings suggested that dietary curcumin is recommended as a prophylactic measure to prevent AFB_1_-induced hepatocellular toxicity and oxidative stress.

## Author Contributions

XZ supervised all the experiments. IM contributed to paper writing. HW, XS, and MH performed the practical work and completed the experiments. XW, ZL, PC, and MAH provided help during experiments.

## Conflict of Interest Statement

MH was employed by Changchun Dirui Medical Company Ltd., No. 3333 liju road, Changchun, China.The other authors declare that the research was conducted in the absence of any commercial or financial relationships that could be construed as a potential conflict of interest.
